# No effects of post-activation performance enhancement in elite male volleyball players under complex training

**DOI:** 10.1038/s41598-024-64604-5

**Published:** 2024-06-14

**Authors:** Sebastian Masel, Marcin Maciejczyk

**Affiliations:** Department of Physiology and Biochemistry, Faculty of Physical Education and Sport, University of Physical Education, Kraków, Poland

**Keywords:** PAPE, Power, Measurements, Accommodating resistance, Strength training, Trap bar deadlift, Physiology, Musculoskeletal system

## Abstract

The aim of this study was to establish reliability of post-activation performance enhancement in three manners: (1) interday morning and afternoon reliability; (2) intraday morning and afternoon reliability; (3) intraday set-to-set reliability. Twelve elite male volleyball players experienced in resistance training performed four identical experimental sessions—two in the morning and two in the afternoon. During each session participants performed a mini complex training session—three sets of a conditioning activity (CA) (3 repetitions of a trap bar deadlift at 80% 1RM with 15% of accommodating resistance) and 90 s after a CA performed squat jump (SJ) with 4 min intra-set rest interval. The ANOVA with repeated measures was used to assess significance of the effect of a CA and ICC to assess reliability of measurements. The PAPE protocol was found to be ineffective to subsequently enhance JH on various occasions. Also, the results of this study suggest that the practitioners may effectively implement appropriately organized complex training as both intraday set-to-set (0.87 and 0.82 for morning sessions; 0.83 and 0.58 for afternoon sessions) and interday morning (0.67) and afternoon (0.8) reliabilities seem to be acceptable. However, introducing two CT sessions within one day is highly questionable as at the moment intraday morning and afternoon reliability is vague (0.88 and 0.48).

## Introduction

Post-activation performance enhancement (PAPE) is a physiological phenomenon that is observed by an increased power output in an explosive exercise such as sprinting or jumping after applying a specific conditioning activity (CA). Potential increases in muscle temperature, muscle and muscle fiber water content and muscle activation have been associated with PAPE effect^1^. Efficacy of PAPE is dependent on introducing an appropriate combination of volume^2,3^ and intensity^3,4^ of a CA and a proper rest interval before implementing an explosive exercise. Different authors of meta-analyses suggest different rest intervals to have the biggest effect: 5–7 min^5^, 6–10 min^6^ or 3–7 min^7^ considering specifically vertical jump performance. PAPE response was found to be highly individual^8^ and self-selected rest intervals can also be effective to acutely enhance performance^9^. Thus, PAPE protocols should be designed appropriately to a given athlete to optimize the training process. Apart from CA attributes, inter-individual differences^10^ and a relative strength level of an individual are also important factors^5,11^ to determine an efficient training protocol.

Current PAPE research indicates that various PAPE protocols may be implemented and acutely enhance subsequent post-CA performance. The authors introduced and found beneficial effects of various application methods such as isometric CA^12,13^, traditional resistance^3,14^, accommodating resistance^15,16^ or flywheel devices^17^. The approach to evaluate PAPE responses may differ between the protocols—the post-CA explosive exercise may be introduced after a single set of a CA^18^, after multiple sets of the same CA^19^ or between each set of the same CA^20^. In order to implement different PAPE protocols with different CAs authors tend to introduce separate experimental sessions^3,18^. After receiving a positive or a negative outcome of the protocol, it is a common practice to omit repeating the same protocol, the researchers simply move to another PAPE protocol and examine the effects of another CA. So far, various jumping tests’ reliability have been examined i.e. countermovement jump (CMJ) and squat jump (SJ)^21^ or drop jump (DJ)^22^. However, no authors put the same interest in PAPE—no study provided results about the reliability of PAPE phenomenon. Using reliable jumping tests to assess post-CA performance is a standard procedure^15,23^, but assessing their effectiveness on various occasions within the same individuals has not been studied so far. As PAPE response is individual^8^, there could be a possibility that the same individual could react differently to the same type of CA. Therefore, one could suggest that repeating the same PAPE protocol may provide different results, especially considering an individual response to a stimulus.

The PAPE phenomenon can be implemented in various manners such as warm-up, testing and monitoring or priming and rewarm-up during the competition^24^. It is also frequently introduced within training methods and is described as a contrast or complex training^25^. These two methods have their differences, but the practitioners tend to use both terms interchangeably, whereas in fact what they introduce to their athletes is complex training (CT)^25^. CT is defined as a training method that involves PAPE—high-load weight training exercise is implemented as a CA and after intra complex recovery interval (ICRI) is alternated with a plyometric or power exercise, set for set^26^. Response to a complex training, similarly to PAPE, was found to be highly individualized and players competing in sports in which jumping actions are crucial for performance may benefit the most to this type of training^26^. Other authors also suggest that CT can be an effective training method to improve vertical jump performance^27,28^. It may be particularly important in relation to professional volleyball as jump demands are high^29^ and increases in jump height may support subsequent effectiveness in offensive actions^30^. Also, volleyball players tend to jump higher than basketball or handball players on high^31^ and college level^32^. Despite the fact that the majority of PAPE research focuses on acute effects of PAPE protocols on various power adaptations^18,20,33^, it cannot be seen in relation to CT. The CT research tends to consider long-term effects of repeatable PAPE incidents on vertical jump or sprinting. The authors put their interest in performance improvements after training interventions lasting ≥ 4 weeks, but they do not consider the acute PAPE effects of CT sessions^25–28^. Marshall et al. analyzed training responses from various CT protocols, but the main focus of these studies was to introduce various PAPE protocols with different CAs^34^. However, they did not analyze acute PAPE responses of multiple CT sessions with the same type of CA. Thus, since no research has focused on the acute PAPE effects of multiple CT sessions with the same CA, it is uncertain if the PAPE effect actually occurs repeatedly within these sessions or it could occur only after training interventions lasting ≥ 4 weeks. It could undermine the reasonableness of introducing CT to the athletes if it cannot be performed regularly for ≥ 4 weeks.

Despite extensive literature regarding PAPE, the current research does not provide interventions regarding the same PAPE protocol with the same CA during separate experimental sessions. Evaluating reliability of the same PAPE protocol within the same sample and in the same conditions may be crucial for further implementation of PAPE phenomenon in athletes’ training programs. It could occur that an experimental protocol which did not enhance subsequent explosive performance may provide different results if it had been retested. Introducing retesting of the same protocol could give a better understanding of PAPE and broaden the application possibilities for the practitioners. PAPE is an individually occuring phenomenon and we wanted to test if it occurs independently from the day and time of the day. Thus, we decided to introduce the same CA under small CT sessions 4 times (2 in the morning and 2 in the afternoon) and examine its reliability. It was hypothesized that the PAPE protocol under CT would have a repeatable enhancement effect on vertical jump performance in elite volleyball players. We also expected an individual response to a CA and introducing an individual analysis was another aim of this study.

## Material and methods

### Study design

**Figure 1:**
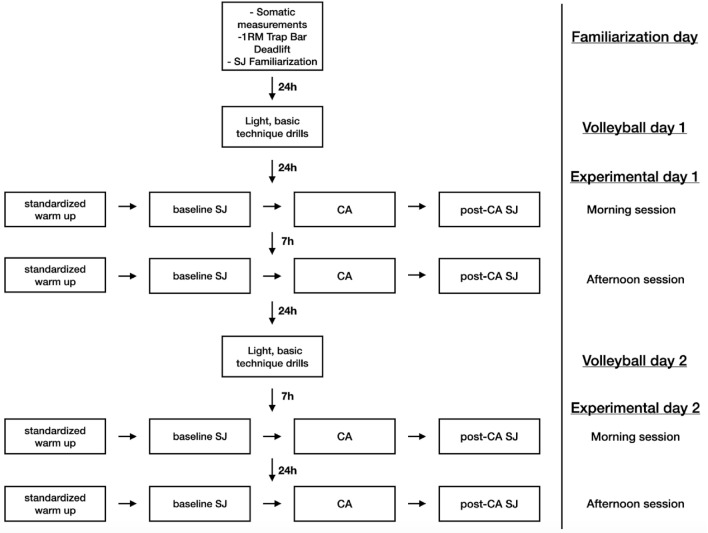
Study Design. 1RM—one repetition maximum; SJ—squat jump; CA—conditioning activity.

This study took three days (familiarization day and two experimental days) and participants took part in five sessions: one familiarization and four experimental sessions (S1, S2, S3 and S4). The familiarization session was performed in the morning (from 10 a.m. to 12 a.m.) and two of the subsequent experimental sessions were performed in the morning (S1 and S3; from 9:30 a.m. to 12:00 a.m.) and two in the afternoon (S2 and S4; from 5:00 p.m. to 7:30 p.m.). Experimental sessions were small CT sessions and were performed in two experimental days and each experimental session took approximately 35 min. The morning and afternoon experimental sessions were performed with approximately 7 h break between the sessions. Apart from experimental days, players participated in their volleyball afternoon training sessions. As this study took place at the beginning of the preparatory cycle, the intensity of volleyball sessions was low, they consisted mainly of basic technique drills and did not involve high intensity activities such as spiking or service. First day of the study was a familiarization session which started with somatic measurements and therefore, a one-rep maximum determination in a trap bar deadlift (1RM) and familiarization with a SJ test were performed. After the familiarization session, the participants were split into four groups of three participants to perform experimental sessions in the same order and avoid potential interruptions during the experimental protocols. In the main part of the study the participants performed four experimental sessions that included a standardized warm-up, baseline SJ, CA (trap bar deadlift with accommodating resistance) and post-CA SJ measurements. A conditioning activity used in the study was 3 repetitions of a trap bar deadlift at 80% 1RM where 65% of 1RM was provided by free weight and approximately 15% of 1RM was accommodating resistance. A conditioning activity was implemented three times during each experimental session—90 s after each CA the participants performed post-CA SJ (Fig. 1.).

Inclusion criteria, required to participate in the study included: (a) professional level of competition (Divisions under Polish Volleyball League (PLS—Polska Liga Siatkówki); (b) valid medical examination to participate in competition; (c) lack of injuries or other health contraindications in the last 6 months. The participants were instructed to maintain their usual dietary and sleep habits throughout the study. They took part in the study voluntarily after being informed about the study protocol and potential risks and benefits of the study and provided informed signed consent. The Bioethics Committee accepted the study protocol (Regional Medical Chamber in Kraków, Poland; opinion no: 1/KBL/OIL/2022) and the study was performed in accordance with the ethical standards of the declaration of Helsinki 2013.

### Participants

Twelve elite male volleyball players (age: 22 ± 2 years; volleyball training experience: 10.2 ± 2.3 years, body height: 193.4 ± 7.6, body mass: 84.1 ± 8.1 kg) experienced in resistance training (7 ± 1.6 years) participated in the study. They practice volleyball daily and compete in the second highest volleyball division in Poland (Tauron 1. League). Volleyball players participating in the study included players competing in every volleyball position: 4 outside hitters, 3 middle blockers, 2 setters, 2 liberos and 1 opposite hitter.

### Warm-up

Each day started with a standardized warm-up that took approximately 15 min and consisted of three parts: (1) aerobic warm-up (2) full-body mobility; (3) dynamic warm-up. To increase body temperature, a standardized warm-up began with 5 min of jogging on a mechanical treadmill at a velocity of 7–8 km/h. Afterwards, full-body mobility was introduced—exercises were performed in 3 positions: quadruped: (a) extending/flexing spine, (b) internal/external rotation in the hips; half-kneeling: (a) adductor mobility, (b) hip mobility in 3 positions, (c) thoracic rotations; plank: (a) isolated downward-upward movement of the scapulas, (b) pushings hips up with straight legs (“downward dog”) and returning to plank position. Last part of the standardized warm-up was a dynamic warm-up that consisted of a set of 10 repetitions each of dynamic stretching exercises: (a) knee to chest with calf raise, (b) heel to buttocks with calf raise. It was followed by (a) 2 sets of pogo jumps (mini jumps using only ankle joints) for 10 s, (b) 5 squat jumps.

### Familiarization session

The familiarization session was conducted in the same manner as previously described^18,35^. It consisted of three parts—somatic measurements, 1RM determination in a trap bar deadlift and familiarization with the SJ test. During somatic measurements body height was measured by a stadiometer (SECA, Germany), whereas body mass and body composition (body fat and lean body mass) were measured using the JAWON scale (Korea, bioelectrical impedance analysis). 1 RM determination was performed after a standardized warm-up and it resulted in the mean relative of 2.07 ± 0.22 kg/body mass. Last part of the session was familiarization with the SJ test and participants executed the SJ test several (3 to 5) times.

### Experimental sessions

**Figure 2:**
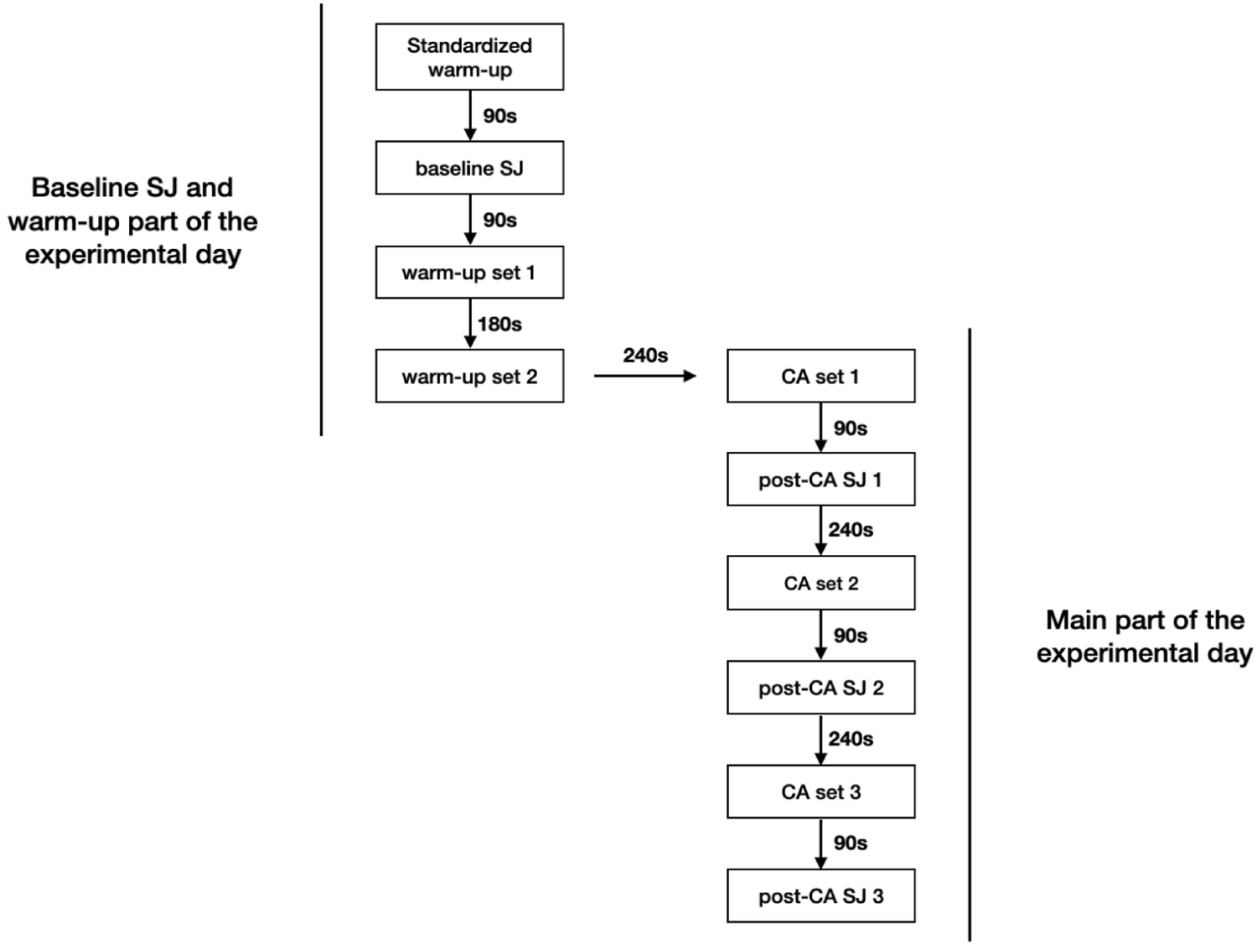
Experimental session flow.

Each experimental session was conducted in the same manner and began with the standardized warm-up and 90 s after the standardized-warm up the participants performed baseline SJ. 90 s after baseline SJ they performed a first warm-up set that consisted of 3 repetitions at 50% of 1RM. 180 s after the first warm-up set the participants performed a second warm-up set that consisted of 3 repetitions at 70% of 1RM. Then, 240 s after the second warm-up set the participants performed a first set of CA of the study—3 repetitions of a trap bar deadlift at 80% of 1RM with approximately 15% of 1RM of elastic bands. 90 s after this set they performed their first post-CA SJ. This cycle of alternating a CA with SJ was repeated two times until the participants performed a CA and post-CA SJ three times in a set for set manner (Fig. 2). In all of the SJ measurements the participants performed two repetitions of SJ and the one with a higher jump height (JH) value was used in further statistical analysis. Additionally, during experimental sessions a velocity monitoring device (VmaxPro/enodePro, Germany) was introduced to increase training motivation and encourage the athletes to put maximum effort during the concentric portion of the lift. VmaxPro was proved to be a reliable and sensitive device for resistance training monitoring and prescription^36^. The athletes were instructed to perform each repetition with a maximal velocity in the concentric phase of the lift and controlled eccentric phase (approximately 2 s of lowering the bar). Due to manufacturer’s instructions on how a trap bar deadlift should be performed to obtain correct measurement data, each repetition was performed dead-stop (full stop at the bottom of the lift) without bouncing the bar off the floor between the repetitions.

### Squat jump measurements

Squat jump measurements were conducted analogously to the previous study by Masel and Maciejczyk^35^. Instructions for the participants included a fast downward movement until they reached approximately 90° of knee flexion, followed by an isometric hold of 2 s and a maximal jump from an isometric position. Isometric hold at the bottom of the squat was counted by the supervisor of the study to optimize a proper execution of the test. OptoJump (Italy) device was used for the measurements—an optical measurement system^37^.

### Band tension measurements

Before the start of the study, band tension was determined with a qualified biomechanist to optimize subsequent measurements during experimental sessions. Adjustments of elastic bands were executed based on previous studies by Wallace et al. and Popp Marin et al.^38,39^. Band tension was determined at different lengths using separately a force plate and a load cell (both produced by a polish manufacturer). Four types of elastic bands (Domyos, Germany) of different thickness were introduced during adjustment and afterwards used throughout the study. Elastic bands were brand new to avoid any potential modifications of band tension.

### Statistical analysis

All data regarding baseline to post-CA changes is presented as mean and standard deviation (SD). Shapiro–Wilk test was used to check the distribution of variables. Anova with repeated measures was implemented to assess the significance of the CA on jumping performance. Levene’s test was used to check the homogeneity of variance within the groups. The differences were considered statistically significant for *p* < 0.05. If ANOVA did not reveal any significant interaction, a post-hoc analysis was not conducted. The effect size (Cohen’s d) was calculated and interpreted as small (0.20), medium (0.50), or large (0.80)^40^. To assess the reliability of PAPE the Intraclass Correlation Coefficient (ICC) was introduced in accordance with guidelines of its use^41,42^. The reliability of the measurements was based on an absolute-agreement, 2-way mixed effects model and interpreted as poor (ICC < 0.5), moderate (0.5–0.75), good (0.75–0.9) and excellent (ICC > 0.9). The reliability was assessed in three manners: 1) interday morning and afternoon reliability; 2) intraday morning and afternoon reliability; 3) intraday set-to-set reliability. The STATISTICA 13.1 PL (StatSoft, Inc., Tulsa, OK, United States) and PQ Stat 1.86 (PQStat Software, Poland) were implemented for statistical calculations.

## Results

### Group analysis

Analyzing the data, no PAPE effect was found in any of the four experimental sessions. Baseline to post-CA changes in JH in SJ were statistically insignificant (Table 1). Baseline JH is constant for different sets within the same experimental session and post-CA performance after different sets is compared with baseline JH for a given session. Also, a consistent small effect size for JH changes was observed (Table 1). ANOVA with repeated measures did not indicate a statistically significant effect for session (F = 0.8; *p* = 0.5; η^2^ = 0.05), set (F = 1.78; *p* = 0.15; η^2^ = 0.04) or interaction between these two factors (F = 0.69; *p* = 0.72; η^2^ = 0.04).

**Table 1:** Results of jumping tests after applicated CA with 90 s rest interval (presented as mean ± SD).

Session	Set	Baseline JH (cm)(95% CI)	post-CA JH (cm)(95% CI)	Cohen’s d
S1	1	45.1 ± 3.0 (43.2 – 47)	44.7 ± 2.9 (42.9 – 46.5)	0.14
	2	45.1 ± 3.0 (43.2 – 47)	44.7 ± 3.5 (42.5 – 46.9)	0.12
	3	45.1 ± 3.0 (43.2 – 47)	44.2 ± 3.0 (42.3 – 46.1)	0.3
S2	1	46.0 ± 2.0 (44.7 – 47.3)	45.9 ± 2.9 (44.1 – 47.7)	0.04
	2	46.0 ± 2.0 (44.7 – 47.3)	45.3 ± 3.6 (43 – 47.6)	0.24
	3	46.0 ± 2.0 (44.7 – 47.3)	45.9 ± 3.0 (44 – 47.8)	0.04
S3	1	44.6 ± 2.6 (43 – 46.2)	44.6 ± 2.7 (42.9 – 46.3)	0
	2	44.6 ± 2.6 (43 – 46.2)	44.4 ± 2.9 (42.6 – 46.2)	0.07
	3	44.6 ± 2.6 (43 – 46.2)	44.8 ± 2.7 (43.1 – 46.5)	0.08
S4	1	46.4 ± 2.8 (44.6 – 48.2)	46.7 ± 3.3 (44.6 – 48.8)	0.10
	2	46.4 ± 2.8 (44.6 – 48.2)	45.6 ± 3.5 (43.4 – 47.8)	0.25
	3	46.4 ± 2.8 (44.6 – 48.2)	45.7 ± 3.9 (43.3 – 48.1)	0.21

JH, jump height.

### Reliability measurements

Table 2 displays the reliability of the measurements in three manner assessment. Interday morning (S1 and S3) reliability was moderate (ICC = 0.67) and afternoon (S2 and S4) was good (0.8). Intraday morning and afternoon reliability was good for S1 and S2 (0.88) and poor for S3 and S4 (0.48). Intraday set-to set reliability was good in morning sessions (S1—0.87 and S3—0.82), whereas in afternoon sessions it was good in S2 (0.83) and moderate in S4 (0.58) (Table 2).

**Table 2:** Reliability of the measurements.

Reliability	Sessions	ICC
Interday morning	1 and 3	0.67
Interday afternoon	2 and 4	0.8
Intraday morning and afternoon	1 and 2	0.88
Intraday morning and afternoon	3 and 4	0.48
Intraday set-to-set	1	0.87
	2	0.82
	3	0.83
	4	0.58

ICC, intraclass correlation coefficient.

### Individual analysis

**Figure 3:**
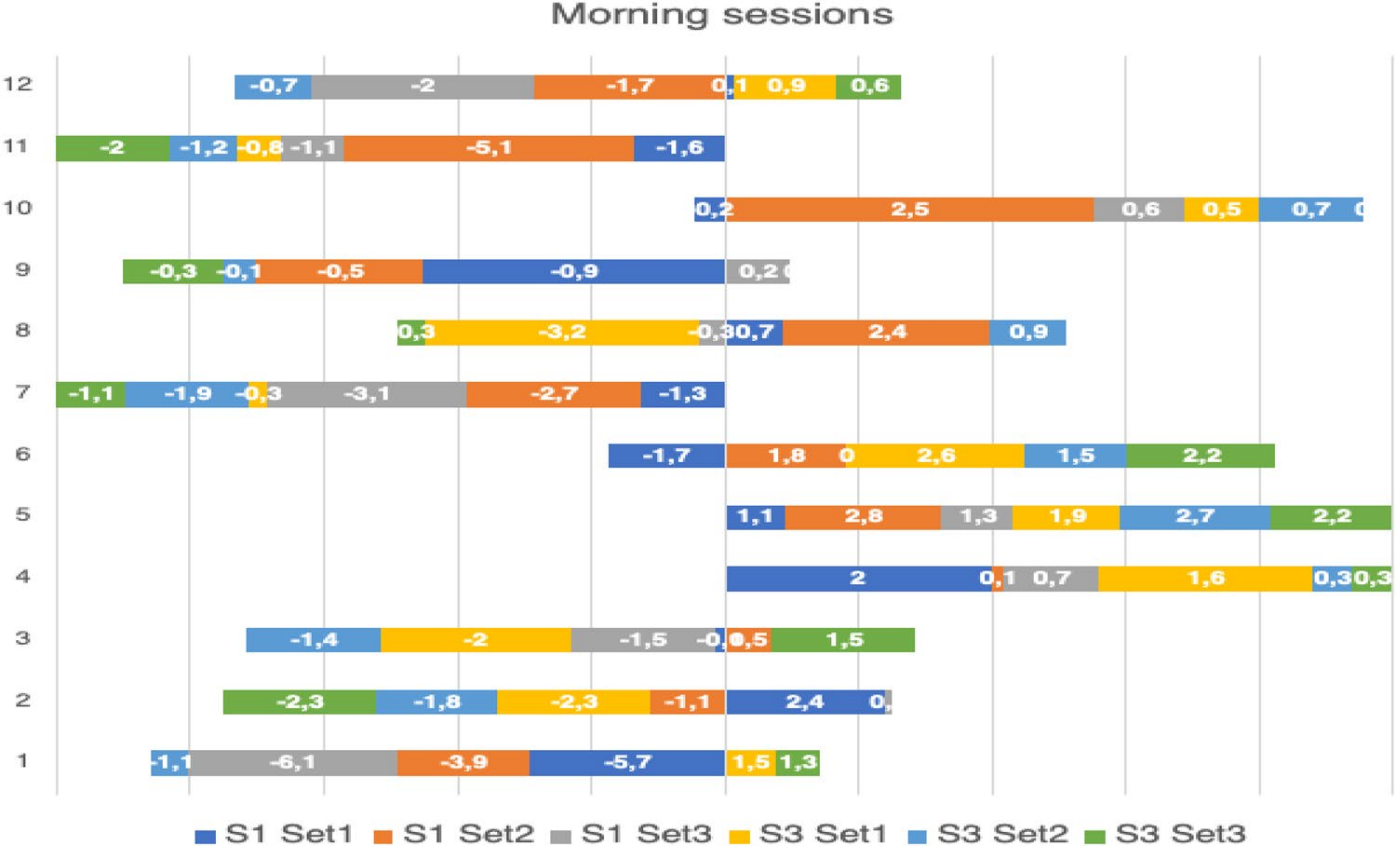
Individual set to set changes in JH (in cm) during morning sessions.

**Figure 4:**
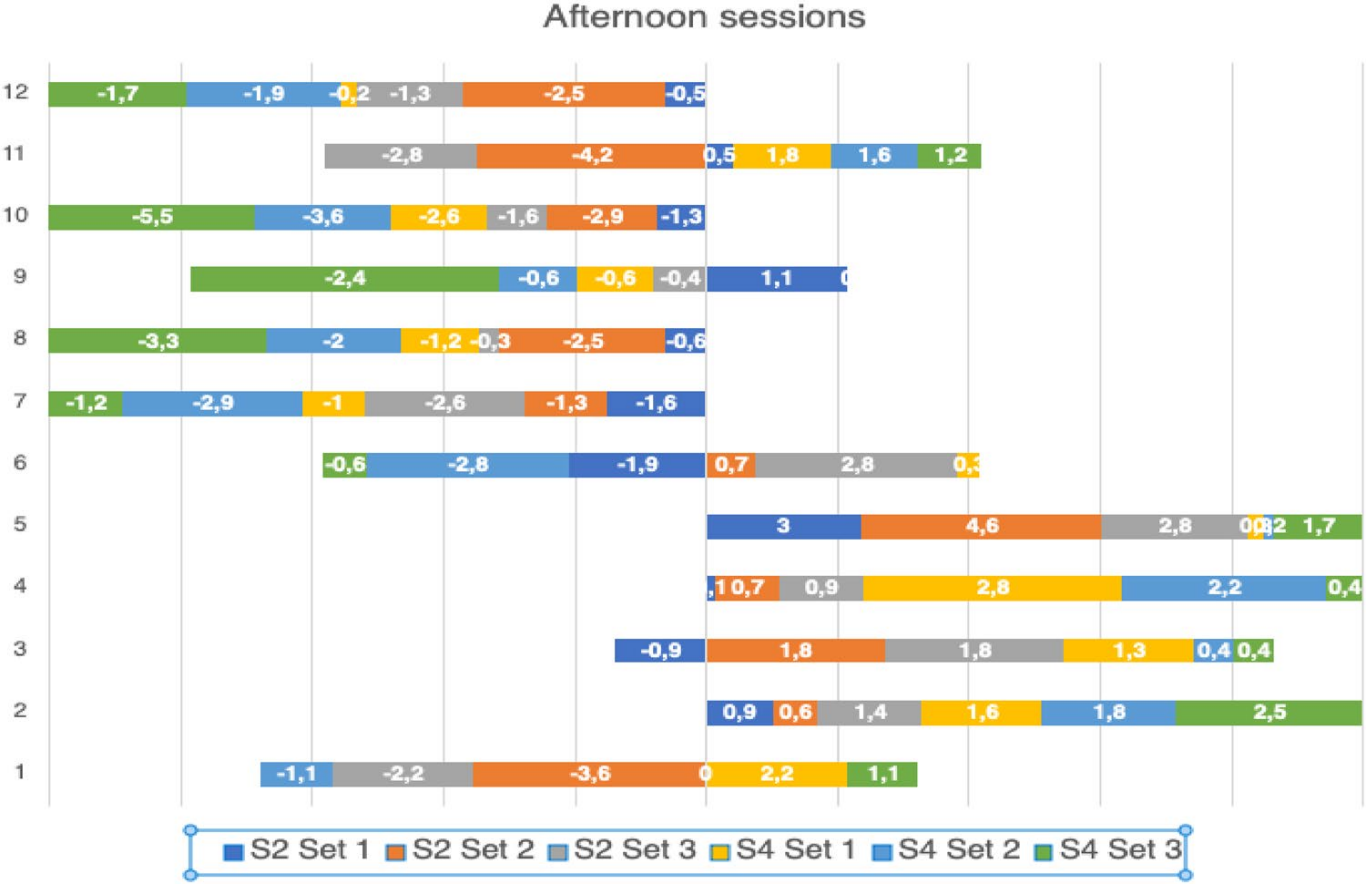
Individual set to set change in JH (in cm) during afternoon sessions.

Individual analysis of the players demonstrates high inter and intraindividual variability of post-CA effects. Despite no statistically significant group effects, some players repeatedly improved their post-CA improvements, whereas the others have mixed or generally negative results (Tables 3 and 4, Figs. 3 and 4).

**Table 3:** Individual analysis of JH changes during morning sessions.

No	Session 1							Session 3						
	Baseline (cm)	Set 1 (cm)	Change in %	Set 2 (cm)	Change in %	Set 3 (cm)	Change in %	Baseline (cm)	Set 1 (cm)	Change in %	Set 2 (cm)	Change in %	Set 3 (cm)	Change in %
12	41.7	41.8	0.2%	40	− 4.1%	39.7	− 4.8%	39.8	40.7	2.3%	39.1	− 1.8%	40.4	1.5%
11	44.4	42.8	− 3.6%	39.3	− 11.5%	43.3	− 2.5%	45.8	45	− 1.7%	44.6	− 2.6%	43.8	− 4.4%
10	42.4	42.2	− 0.5%	44.9	5.9%	43	1.4%	43	43.5	1.2%	43.7	1.6%	43	0.0%
9	48.8	47.9	− 1.8%	48.3	− 1.0%	49	0.4%	48	48	0.0%	47.9	− 0.2%	47.7	− 0.6%
8	43.1	43.8	1.6%	45.5	.,6%	42.8	− 0.7%	44.6	41.4	− 7.2%	45.5	2.0%	44.3	− 0.7%
7	43.5	42.2	− 3.0%	40.8	− .,2%	40.4	− 7.1%	42.1	41.8	− 0.7%	40.2	− 4.5%	41	− 2.6%
6	42.2	40.5	− 4.0%	44	4.3%	42.2	0.0%	41.5	44.1	6.3%	43	3.6%	43.7	5.3%
5	48.8	49.9	2.3%	51.6	5.7%	50.1	2.7%	47.7	49.6	4.0%	50.4	5.7%	49.9	4.6%
4	42.1	44.1	4.8%	42.2	0.2%	42.8	1.7%	42.7	44.3	3.7%	43	0.7%	43	0.7%
3	47.1	47	− 0.2%	47.6	1.1%	45.6	− 3.2%	45.4	43.4	− 4.4%	44	− 3.1%	46.9	3.3%
2	46.4	48.8	5.2%	45.3	− 2.4%	46.5	0.2%	47.9	45.6	− 4.8%	46.1	− 3.8%	45.6	− 4.8%
1	50.7	45	− 11.2%	46,8	− 7.7%	44.6	− 12.0%	46.4	47.9	3.2%	45.3	− 2.4%	47.7	2.8%
x	45.1	44.7	− 0.9%	44.7	− 0.8%	44.2	− 2.0%	44.6	44.6	0.2%	44.4	− 0.4%	44.8	0.4%
SD	3.0	2.9	4.2%	3.5	5.5%	3.0	4.1%	2.6	2.7	3.9%	2.9	3.0%	2.7	3.1%

**Table 4:** Individual analysis of JH changes during afternoon sessions.

No	Session 2							Session 4						
	Baseline (cm)	Set 1 (cm)	Change in %	Set 2 (cm)	Change in %	Set 3 (cm)	Change in %	Baseline (cm)	Set 1 (cm)	Change in %	Set 2 (cm)	Change in %	Set 3 (cm)	Change in %
12	43.5	43	− 1.1%	41	− 6.1%	42.2	− 3.1%	41	40.8	− 0.5%	39.1	− 4.9%	39.3	− 4.3%
11	46.2	46.7	1.1%	42	− 10.0%	43.4	− 6.5%	45.5	47.3	3.8%	47.1	3.4%	46.7	2.6%
10	45	43.7	− 2.9%	42.1	− 6.9%	43.4	− 3.7%	46.7	44.1	− 5.9%	43.1	− 8.4%	41.2	− 13.3%
9	47.1	48.2	2.3%	47.1	0.0%	46.7	− 0.9%	48	47.4	− 1.3%	47.4	− 1.3%	45.6	− 5.3%
8	45.5	44.9	− 1.3%	43	− 5.8%	45.2	− 0.7%	47.3	46.1	− 2.6%	45.3	− 4.4%	44	− 7.5%
7	45	43.4	− 3.6%	43.7	− 3.0%	42.4	− 6.1%	43	42	− 2.4%	40.1	− 7.2%	41.8	− 2.9%
6	43	41.1	− 4.4%	43.7	1.6%	45.8	6.1%	46.1	46.4	0.6%	43.3	− 6.5%	45.5	− 1.3%
5	48	51	6.3%	52.6	8.7%	50.8	5.5%	49.9	50.2	0.6%	50.1	0.4%	51.6	3.3%
4	43.4	43.5	0.2%	44.1	1.6%	44.3	2.0%	43.1	45.9	6.1%	45.3	4.9%	43.5	0.9%
3	49.7	48.8	− 1.8%	51.5	3.5%	51.5	3.5%	49.7	51	2.5%	50.1	0.8%	50.1	0.8%
2	47.3	48.2	1.9%	47.9	1.3%	48.7	2.9%	45.5	47.1	3.4%	47.3	3.8%	48	5.2%
1	48	48	0.0%	44.4	− 8.1%	45.8	− 4.8%	50.4	52.6	4.2%	49.3	− 2.2%	51.5	2.1%
x	46.0	45.9	− 0.3%	45.3	− 1.9%	45.9	− 0.5%	46.4	46.7	0.7%	45.6	− 1.8%	45.7	− 1.6%
SD	2.0	2.9	2.8%	3.6	5.4%	3.0	4.2%	2.8	3.3	3.3%	3.5	4.3%	3.9	5.1%

## Discussion

The most important finding of this study is that the reliability of PAPE phenomenon under mini CT sessions in elite volleyball players may be considered relatively good. Our study proved that the similar effect can occur independently from day and time of the day. We did not observe the phenomenon occuring in the first session and it also did not occur during subsequent sessions. 5 out of 8 ICC measurements showed good reliability, 2 out of 8 moderate reliability and only 1 out of 8 poor reliability. PAPE effects seem to be more reliable during morning sessions as 3 out of 4 ICC measurements indicated good reliability and 1 out of 4 moderate reliability whereas during afternoon sessions 2 of 4 showed good reliability, 1 out of 4 moderate reliability and 1 out of 4 poor reliability (Table 2) Additionally, the measurements of SJ were similar at the baseline and after introducing a CA for multiple sets during the sessions performed at the same time of the day (Table 1).

To our knowledge, this is the first study that examined the reliability of PAPE effects under CT. This type of training, using PAPE phenomenon, is often introduced to the athletes to develop lower body force, velocity, power and jump height^34^. Freitas et al. suggested that this type of training can be especially beneficial for players competing in sports in which jumping actions are crucial for performance^26^. Also, high vertical JH may allow to maximize the efficacy of volleyball offensive actions^30^. In comparison with basketball or handball, the volleyball players tend to jump higher^31,32^ and basketball or handball also involve a lot of running and changes of direction that is not observed in volleyball. Introducing this type of training to the volleyball players may be absolutely relevant as the majority of their technical elements (spiking, blocking, service, setter’s setting) is based on jumping. Thus, in this study we proposed the following procedure: introducing the same PAPE protocol 4 times (2 sessions in the morning, 2 in the afternoon) under small CT sessions. We decided to implement a CA to elite volleyball athletes that we already used previously^18,23,35^ and had an individual response and a potentiating effect on strong individuals^18,35^. Apart from examining the reliability of PAPE, introducing sessions at different times of the day provides additional benefit as this area of PAPE research has not been studied extensively so far^43,44^. Additionally, our study provided an important insight into PAPE research regarding elite male volleyball players as so far, the majority of evidence has focused on elite female volleyball players^45–47^.We decided to investigate this matter in elite volleyball players with high relative strength level (relative 1RM in a trap bar deadlift 2.07 ± 0.22 kg/body mass) as it was suggested that the athletes with relative strength level of > 1.5 kg/body mass^5^ or > 2 kg/body mass^11^ should be a target group for this kind of measurements. Examining the reliability of CT in such population may be valuable as its results can be directly applicated into sports training.

The results of our study indicate an overall good reliability of PAPE effects under CT in elite volleyball players. Interday reliability of morning (0.67) and afternoon (0.8) sessions suggests that there is a high likelihood that the same training protocol introduced within the same population on different training days could generate similar training effects. Intraday reliability between morning and afternoon sessions on the same day of 0.88 for S1 and S2 and 0.48 for S3 and S4 is ambiguous and does not really provide a meaningful insight. A discrepancy between two days is very high and these kinds of results suggest that on one day while introducing CT twice, we can expect the same training effects and on a different day we could obtain a totally different outcome. Third reliability measurements concerned set-to-set reliability to check if PAPE effects may be reliable within the same training session when multiple sets of CA and an explosive exercise are introduced. ICC results of 0.87 and 0.83 for morning sessions and 0.82 and 0.58 for afternoon sessions indicate that we can expect a similar training effect within the same training session. Therefore, there is a high likelihood that if an individual experiences a potentiating effect after the first set of a CA, the effect may be sustained after completing subsequent sets of a CA. That is an important conclusion for the practitioners as they usually tend to program multiset training plans of a given exercise to their athletes. If the effect was not reliable over multiple sets, it would decrease the efficacy and utility of this type of training.

Introducing ICC to assess the reliability of the measurements was our primary goal for this study but monitoring pre to post-CA changes in JH and its significance could be another method to check the reliability of PAPE effect. In this manner, the results are consistent—100% of the sets performed in this study indicated no statistical significance in JH changes, the protocol was consistently ineffective for the group. It shows that a training protocol introduced to the volleyball players was ineffective (considering results for the group) and should not be subsequently transferred to their training program. One could speculate if ICC reliability measurements are valuable as no PAPE effects were found within the group after any set in four experimental sessions. However, despite not having significant pre to post-CA changes in JH, the results of this study still provide important conclusions about reliability of the phenomenon. After this kind of investigation, a decision-making process for the coach becomes less complicated as he can reject this training protocol for the training group as there is a high likelihood that the same training protocol will not provide different results. This allows to program the training process more efficiently as the coach can quickly adjust a training program and not implement inefficient training protocol to the group continuously. It is especially important in a group setting where proper time management is particularly important as the coaches usually have limited time for the session.

Despite not seeing a potentiating effect for the group, it is worth introducing an individual analysis of the players as both PAPE effect and response to a CT are individual^8,26^. So far, no guidelines have been proposed on how to classify PAPE responders and non-responders. As the reliability of PAPE has not been studied yet, we propose an arbitral classification to assess an individual reliability to a training protocol. In this study, we obtained 12 pre to post-CA changes in JH and we suggest a following classification: (1) ≤ 4 positive changes (up to 33% of sets) in post-CA JH—a reliable non-responder; (2) 5–8 positive changes (41.7–66.7% of sets) in post-CA JH—ambiguous results, cannot classify clearly; (3) ≥ 9 positive changes in post-CA JH (75–100% of sets)—a reliable responder. Taking into consideration the above mentioned classification, it can be seen that 7 out of 12 players would be classified as reliable non-responders, 2 out of 12 reliable responders and 3 out of 12 cannot be clearly classified. An interesting fact is that 1 participant (n7) did not improve his post-CA SJ after any of 12 sets and 2 participants (n4 and n5) improved their post-CA SJ after all of 12 sets (Figs. 3 and 4). Despite the classification being arbitrary, we would recommend the coaches to test post-CA performance in their athletes and then decide if the CA is appropriate for a given individual. Therefore, after testing the athlete multiple times and having post-CA improvements in performance on ≥ 75% occasions, the training protocol is probably efficient and can be used to increase athlete’s performance. On the contrary, if the athlete cannot achieve this percentage using a given training protocol, he should probably be introduced to a different protocol (if he had had ambiguous results) or even a different training method (if he had been a reliable non-responder).

An individual analysis provided an insight regarding their individual response to a CA stimulus but it can be also analyzed in relation to their playing position. It is an important concern as the authors found a large variability in jump demands between professional volleyball players on different positions during training sessions and matches^29,48^.The setters generally have the highest number of the jumps but their jumps are relatively low in relation to their maximal jump height (approximately 56%). Also, their weekly training load is the highest as the specificity of their position involves a high number of jumps^29^. Middle blockers also have a high number of the jumps as apart from technical elements such as block, spiking and service, their actions also involve simulation of the spike^48^ and their relative jump height is higher than the setters (approximately 64%). Opposites, in comparison with the middle blockers, are generally introduced to a similar jumping load during the matches^29,48^ but their number of the jumps during the week is lower and their relative jump height is the highest of all positions (approximately 73%). The outside hitters’ jumping load seems to be the smallest as their number of the jumps during the matches and weekly sessions is the lowest of all positions^29,48^. Our results provided a different PAPE response based on players’ position. One of the setters (n1), liberos and outside hitters (n12-n7) were found to be reliable non-responders, two of the middle blockers (n5, n4) were found to be reliable responders, and the results of the second setter (n2), the third middle blocker (n6) and the opposite (n3) were ambiguous and cannot be clearly classified. Therefore, apart from the general individual response to an applied CA, an individual response based on players’ position can also be observed. Based on this analysis, the middle blockers seem to be the most appropriate to apply this type of intervention as their efficacy is the highest of all positions.

Our study provides practical applications for the coaches regarding PAPE phenomenon under CT that was examined to be reliable, especially during the same training session. Also, we proposed a classification that can be applied in the same manner or slightly modified by a coach and simplify the decision making process about training methods for the athlete. Training intervention was introduced to professional athletes so the results of the study can be easily transferred to athletes’ training programs. However, we would like to see our results reexamined in the group that positively responds to an introduced CA. Also, despite examining elite volleyball athletes we suggest applying results of this study with caution as this is the first study that examined the reliability of PAPE response. We recommend to test the athlete’s response to a given CA several times and then adjust the training process as a response can vary drastically between the athletes within the same sport. We cannot assume with certainty that further investigation will confirm our results as the PAPE research is broad and the athlete’s response to a given CA can be unpredictable. Thus, in future research the investigators should focus on repeating the same training protocols with various application methods (i.e. isometric, flywheel, accommodating resistance) and check its reliability instead of introducing new training protocols and only changing rest intervals or volume and intensity of a CA.

## Limitations of the study

Despite its strengths, the study also has a few limitations. Firstly, the study involved only twelve players so in the future studies the number of participants could be increased. However, it was necessary to include in the study only players performing the same training (from the same team) to avoid the effect of the variety of training performed. Also, we tested the reliability of only one protocol, investigators could consider introducing a few training protocols and testing its reliability. Regarding results of our study, one could question introducing the same protocol with the same CA and testing its reliability when its initial effects did not provide PAPE effect. Thus, in future research, investigators could consider testing reliability of these procotols that had a potentiating effect in the group.

Additionally, we would recommend implementing force plates for the extended measurements of the jumps. That would allow to not only assess JH but also kinetic characteristics of the jump i.e. depth and velocity of the jump that affect subsequent JH^49,50^. It would be particularly valuable if the investigators decided to introduce countermovement jump as a baseline and post-CA explosive exercise, that has no isometric pause at the bottom as SJ. Then, it could be assessed if an athlete repeatedly increases his post-CA performance due to a PAPE effect or possibly due to changing execution of the jump.

## Conclusions

This study provides a novel understanding of the PAPE phenomenon under CT in elite male volleyball players. The PAPE protocol was found to be ineffective to subsequently enhance JH on various occasions. Results of this study also suggest that the practitioners may effectively implement appropriately organized CT as both intraday set-to-set and interday morning and afternoon reliabilities seem to be acceptable. However, they should seek other CAs as the one used in this study was not appropriate to induce PAPE response. Implementing CT at both times of the day may be beneficial with a small advantage of afternoon sessions. Introducing two CT sessions within one day is highly questionable as at the moment intraday morning and afternoon reliability is vague.

## Data Availability

The datasets analyzed during the study are available from the corresponding author (SM) on reasonable request.
